# Obituary: Dr Dimitri Tassiopoulos

**DOI:** 10.1080/17290376.2017.1337329

**Published:** 2017-06-23

**Authors:** Nancy Phaswana-Mafuya

**Affiliations:** ^a^ Department of Sociology, University of Central Florida, Orlando, USA


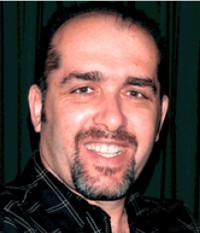


Dimitri managed *SAHARA-J: Journal of Social Aspects of HIV/AIDS* exceptionally well and ensured its swift transition to Scholar One. He did so with great enthusiasm and passion right up to him being diagnosed with a debilitating illness that led to his untimely passing. Dimitri diligently set up and refined the internal systems, structures, processes and marketing tools for the journal’s effective and efficient management and was instrumental in acquiring higher impact factor for the journal. Dimitri’s death is a great loss, not only for SAHARA J but also on a personal level as he had become an all-time friend, my confidant and soul brother.

